# What barriers and facilitators influence the implementation of infrared vein visualization technology for pediatric peripheral intravenous catheterization? A TDF-informed qualitative study

**DOI:** 10.3389/fped.2026.1933634

**Published:** 2026-10-29

**Authors:** Wenjing Song, Hongzhan Jiang, Lu Chen, Lili Liu

**Affiliations:** 1 Department of Nursing, Capital Center for Children’s Health, Capital Medical University, Capital Institute of Pediatrics, Beijing, China; 2 School of Nursing, Beijing University of Chinese Medicine, Beijing, China

**Keywords:** barriers, facilitators, infrared vein visualization, pediatric nursing, peripheral intravenous catheterization, qualitative study, theoretical domains framework

## Abstract

**Background:**

Infrared vein visualization technology is recommended by the Infusion Nurses Society for pediatric peripheral intravenous catheterization in patients with difficult venous access. Despite evidence supporting its clinical efficacy, routine adoption in pediatric nursing practice remains limited. Understanding the barriers and facilitators to implementation is essential for bridging the evidence–practice gap.

**Objective:**

To systematically identify and analyze the barriers and facilitators influencing the implementation of infrared vein visualization technology for peripheral intravenous catheterization in pediatric nurses, using the Theoretical Domains Framework (TDF) as an analytical lens.

**Methods:**

A qualitative descriptive study was conducted at a tertiary children's specialist hospital in Beijing, China. Fifteen pediatric nurses from internal medicine, surgery, emergency, and intensive care units were purposively sampled between September and October 2024. Semi-structured interviews were conducted, guided by an interview schedule developed according to the 14 domains of the refined TDF. Data were analyzed using a TDF-informed framework analysis involving deductive and inductive coding.

**Results:**

Nine barriers and five facilitators were identified across seven TDF domains: knowledge, skills, social/professional role and identity, goals, beliefs about consequences, environmental context and resources, and social influences. Key barriers included insufficient vascular imaging knowledge, inadequate technical competence, lack of standardized operating procedures, uncertainty regarding professional role boundaries, dependence on conventional cannulation practices, perceived procedural complexity, distrust of device accuracy, lack of pediatric-specific devices, and interference from children's crying and restlessness. Facilitators included enhancement of core professional competence, improved success rates in difficult intravenous access, enhanced puncture efficiency in emergency settings, improved healthcare experience and reduced resource consumption, and positive peer demonstration effects. Three overarching themes were synthesized: system-level implementation conditions require optimization, practice-level willingness among nurses requires enhancement, and outcome-level sustainability needs strengthening.

**Conclusion:**

The implementation of infrared vein visualization technology in pediatric nursing is influenced by multi-level factors spanning individual, organizational, and environmental dimensions. These findings may inform the development and evaluation of targeted, theory-informed intervention programs that address the identified barriers while leveraging the facilitators, with the aim of promoting evidence-based technology adoption and improving the quality of pediatric intravenous therapy.

## 1 Introduction

Peripheral intravenous catheterization (PIVC) is one of the most frequently performed invasive procedures in hospitalized children and is essential for the administration of fluids, medications, blood products, contrast agents, and other intravenous therapies (1). Despite its routine nature, successful PIVC insertion in pediatric patients remains technically challenging. Children often have smaller, less visible, and less palpable veins, and venous access may be further complicated by younger age, prematurity, dehydration, obesity, edema, chronic illness, previous venipuncture, and anxiety-related movement during the procedure (2, 3). The Difficult Intravenous Access (DIVA) score was developed to identify children at increased risk of failed first-attempt insertion, incorporating factors such as prematurity, young age, and vein visibility and palpability (3). Failure to obtain intravenous access on the first attempt is not merely a technical inconvenience; repeated attempts are associated with pain, distress, delayed treatment, loss of trust, increased workload, and additional healthcare costs (4, 5). International evidence also indicates considerable variation in pediatric PIVC practice and performance, underscoring the need for strategies that improve first-attempt success and minimize preventable harm (1).

Infrared or near-infrared vein visualization technology has been introduced as an adjunctive strategy to improve peripheral venous access. These devices use near-infrared light to detect hemoglobin absorption and project or display the course of superficial subcutaneous veins, thereby assisting clinicians in identifying potentially suitable cannulation sites. In pediatric settings, where veins may be difficult to visualize or palpate, this technology has been proposed to improve cannulation success and the experience of children, families, and clinicians. Randomized trials have reported improved first-attempt success in selected subgroups, such as children with high DIVA scores or critical illness (6, 7), whereas other trials and systematic reviews found no consistent benefit across unselected pediatric populations (8, 9). These mixed findings suggest that the technology is unlikely to be a universal solution and that its value depends on patient selection, workflow integration, and local clinical context.

The inconsistent translation of infrared vein visualization technology into routine pediatric practice highlights a broader implementation problem. The successful adoption of medical technologies is influenced not only by clinical efficacy but also by pediatric nurses' knowledge, skills, beliefs, habits, professional roles, environmental resources, organizational culture, and social influences. In busy pediatric wards, emergency departments, and infusion units, decisions about whether and when to use vein visualization devices may be shaped by time pressure, device availability, perceived reliability, prior experience, confidence in traditional cannulation skills, training opportunities, infection-control considerations, and expectations from parents or colleagues. Thus, understanding the barriers and facilitators perceived by frontline nurses is crucial for designing implementation strategies that are feasible, acceptable, and sustainable.

The Theoretical Domains Framework (TDF) provides a comprehensive theory-informed approach for identifying determinants of healthcare professional behavior and implementation problems. The refined TDF comprises 14 domains: knowledge, skills, social/professional role and identity, beliefs about capabilities, optimism, beliefs about consequences, reinforcement, intentions, goals, memory, attention and decision processes, environmental context and resources, social influences, emotion, and behavioral regulation (10). It has been widely used in implementation research to explore why evidence-based practices are or are not adopted and to inform the development of targeted behavior-change interventions (11, 12). Applying the TDF to the implementation of infrared vein visualization technology can therefore move the research focus beyond whether the technology “works” under trial conditions toward understanding how, for whom, and under what circumstances it can be incorporated into pediatric PIVC practice.

To date, most studies of infrared vein visualization technology in children have focused on quantitative outcomes such as first-attempt success, number of attempts, procedure time, and pain scores. Less is known about the experiences, perceptions, and contextual factors that influence clinicians' use of this technology in everyday pediatric care. This represents an important evidence gap because implementation failure may occur even when a technology is available and potentially beneficial. Therefore, this TDF-informed qualitative study aimed to explore pediatric nurse' perceived barriers and facilitators to implementing infrared vein visualization technology for pediatric peripheral intravenous catheterization. By identifying modifiable determinants of use, this study seeks to inform context-sensitive implementation strategies that optimize technology adoption, improve vascular access practice, and reduce the burden of repeated cannulation attempts for children and families.

## 2 Methods

### 2.1 Study design

This was a qualitative descriptive study informed by the Theoretical Domains Framework (TDF). Semi-structured interviews were conducted to explore nurses' perceived barriers and facilitators to the implementation of infrared vein visualization technology for pediatric peripheral intravenous catheterization. The TDF was used to guide the development of the interview topic guide and to support data coding and interpretation. The study was reported in accordance with the Consolidated Criteria for Reporting Qualitative Research (COREQ) checklist (13).

### 2.2 Study setting

The study was conducted between September and October 2024 in a tertiary grade A children's specialist hospital in China. The hospital provides comprehensive pediatric medical services, including general pediatric medicine, pediatric surgery, emergency care, and pediatric intensive care. Infrared vein visualization technology had been introduced into clinical practice in the hospital to assist peripheral intravenous catheterization in children, particularly in patients with difficult intravenous access. However, the extent and pattern of its use varied across clinical departments and individual nurses.

### 2.3 Participants and sampling

Purposive sampling was used to recruit nurses from the departments of internal medicine, surgery, emergency medicine, and intensive care medicine. These departments were selected because peripheral intravenous catheterization is frequently performed in these clinical settings, and nurses in these areas are likely to have direct experience with pediatric vascular access and the use or non-use of infrared vein visualization technology.

Eligible participants were registered nurses who: (1) were currently working in one of the selected departments; (2) had been engaged in pediatric clinical nursing practice for at least five years; (3) had experience performing pediatric peripheral intravenous catheterization; (4) had knowledge of, experience with, or potential access to infrared vein visualization technology in clinical practice; and (5) were willing to participate and provide written informed consent.

Nurses were excluded if they were: (1) in a rotation, internship, or training period and not independently responsible for pediatric intravenous catheterization; (2) absent from clinical work during the study period; or (3) unwilling or unable to complete the interview.

To capture diverse perspectives, maximum variation was considered during sampling in terms of department, years of nursing experience, professional title, educational background, and experience with infrared vein visualization technology. Recruitment continued until data saturation was achieved, defined as the point at which no new themes, categories, or meaningful insights emerged from the interviews. A total of 15 nurses were ultimately included in the study. A sampling matrix combining these characteristics was developed, and potential participants were approached through departmental nurse managers or by direct invitation; the matrix was reviewed iteratively to ensure representation of each department, professional title group, and level of technology exposure. All participants ultimately held bachelor's degrees and had at least five years of pediatric nursing experience, reflecting the hospital's staffing profile for registered nurses in the participating units; within these constraints, variation was sought in department, professional title, years of experience, and technology exposure. Recruitment continued until data saturation was achieved, defined as the point at which no new themes, categories, or meaningful insights emerged from the interviews. Saturation was assessed iteratively after each interview by the interviewer and a second researcher; when the fifteenth interview yielded no new codes, categories, or themes, recruitment was stopped. A total of 15 nurses were ultimately included in the study.

### 2.4 Development of the interview guide

A semi-structured interview guide was developed based on the 14 domains of the Theoretical Domains Framework (TDF), relevant literature on pediatric peripheral intravenous catheterization and infrared vein visualization technology, and discussions within the research team. The interview guide was designed to explore nurses' knowledge, skills, decision-making processes, emotional responses, beliefs, perceived environmental and resource-related constraints, social influences, intentions, goals, reinforcement, and behavioral regulation related to the implementation of infrared vein visualization technology.

The interview guide consisted of six modules: knowledge, skills, and decision-making processes; emotions and beliefs; environmental context, resources, and the nature of the behavior; social influences and team climate; goals, intentions, reinforcement, and behavioral regulation; and summary and additional comments. The full interview guide, including the mapping of interview questions to the 14 TDF domains, is provided in Supplementary Table S1.

The preliminary guide was reviewed by experts in pediatric nursing, vascular access, qualitative research, and implementation science to ensure its relevance, clarity, and theoretical alignment with the TDF. The interview guide was piloted with two nurses to assess the comprehensibility and flow of the questions. Minor wording revisions were made according to the pilot feedback; the scope, content, and TDF coverage of the questions were not materially altered. The two pilot participants were recruited separately and were not included in the final sample of 15 nurses; their data were therefore not included in the final analysis.

### 2.5 Data collection

Potential participants were approached by the research team through departmental nurse managers or direct invitation. The purpose, procedures, voluntary nature of participation, and confidentiality measures were explained before the interview. Written informed consent was obtained from all participants.

Individual semi-structured interviews were conducted between September and October 2024. Interviews were carried out in a quiet and private room within the hospital or via online format according to participant preference and work schedule. Each interview lasted approximately 40–50 min. All interviews were conducted by WJS, who had experience in qualitative interviewing and was not directly involved in the participants' clinical performance evaluation. WJS is a pediatric nurse researcher with training in qualitative methods and clinical experience in pediatric intravenous access. Before the study, WJS had clinical exposure to infrared vein visualization devices and held a generally positive assumption about their potential value for difficult venous access; this assumption was acknowledged, discussed within the research team, and bracketed during coding and interpretation. WJS had no managerial or evaluative authority over participants, and the researcher-participant relationship was professional; participants were informed that their participation would not affect their clinical duties or performance evaluation. During the interviews, participants spontaneously discussed both infrared vein visualization and ultrasound-guided vascular access, comparing the two modalities and reflecting on their professional boundaries; such comparisons were probed when relevant to the research questions.

With participants' permission, interviews were audio-recorded. Field notes were taken during and immediately after each interview to document non-verbal cues, contextual information, and the interviewer's preliminary reflections. The audio recordings were transcribed verbatim within 24 h after each interview. To ensure accuracy, transcripts were checked against the original recordings by two researchers. All identifiable personal information was removed, and each participant was assigned a unique code, such as N1, N2, and N3.

### 2.6 Data analysis

Data analysis was conducted concurrently with data collection to allow emerging findings to inform subsequent interviews. A TDF-informed framework analysis approach was used (14). The analysis involved both deductive and inductive coding. Deductive coding was guided by the 14 TDF domains, while inductive coding allowed new themes and subthemes to emerge from the data.

The analysis proceeded through the following steps:

1. Familiarization: Two researchers independently read the transcripts repeatedly to become immersed in the data and to gain an overall understanding of participants’ experiences.
2. Initial coding: Meaningful text segments related to barriers and facilitators were identified and coded. Initial codes were assigned deductively to relevant TDF domains where appropriate. Data that did not fit existing domains were coded inductively. When a data segment appeared relevant to more than one TDF domain, the research team discussed it and assigned it to the domain judged most salient to the participant's account; segments carrying distinct meanings across domains could be assigned to more than one domain. Although the interview guide covered all 14 TDF domains, only the domains in which participants substantively articulated barriers or facilitators to implementation are reported in the findings; the remaining domains did not emerge as distinct determinants in this dataset.
3. Development of an analytical framework: The research team compared and discussed initial codes, refined code definitions, and developed a coding framework.
4. Indexing and charting: All transcripts were coded using the agreed framework. Codes were organized within and across TDF domains to identify patterns, similarities, and differences among participants and departments.
5. Theme generation and interpretation: Codes were grouped into subthemes and overarching themes representing key barriers and facilitators to implementation. The research team discussed the relationships between themes and considered how these findings could inform future implementation strategies. The nine barriers and five facilitators were then synthesized at a higher level of abstraction into three overarching themes, grouped according to the level at which implementation change is required (system, practice, and outcome levels); this synthesis was iterative and discussed within the research team.

To enhance analytical rigor, at least two researchers independently coded a subset of transcripts. Discrepancies were resolved through discussion until consensus was reached. When necessary, a third researcher was consulted. Data management and coding were supported by NVivo 12.0 software.

### 2.7 Trustworthiness

Several strategies were used to ensure the trustworthiness of the study. Credibility was enhanced through purposive sampling across multiple departments, in-depth interviews, and investigator triangulation during data coding and interpretation. The research team held regular meetings to discuss emerging findings, challenge assumptions, and refine interpretations. Dependability was supported by maintaining an audit trail, including interview guides, field notes, coding decisions, and analytical memos. Confirmability was strengthened through reflexive discussions among researchers to identify and minimize the influence of prior assumptions regarding infrared vein visualization technology. Transferability was supported by providing detailed descriptions of the study setting, participants, sampling strategy, and implementation context.

### 2.8 Ethical considerations

This study was conducted in accordance with the principles of the Declaration of Helsinki. Ethical approval was obtained from the Ethics Committee of the participating hospital, with the approval number SHERLL2024005. All participants were informed of the study purpose, procedures, potential risks and benefits, confidentiality measures, and their right to withdraw at any time without penalty. Written informed consent was obtained directly from each participant (all participants were adult registered nurses) before data collection. All interview recordings and transcripts were anonymized and stored securely. Only members of the research team had access to the data.

## 3 Results

### 3.1 Participant characteristics

A total of 15 pediatric nurses participated in the semi-structured interviews. Data saturation was reached after the fifteenth interview, as no new codes, categories, or themes emerged. Participants were coded from N1 to N15 to protect confidentiality. All participants were female. Their ages ranged from 27 to 43 years, with a mean age of 34.9 years. Their pediatric clinical nursing experience ranged from 6 to 21 years, with a mean of 12.2 years. Fourteen participants held a bachelor's degree and one held a postgraduate degree. Seven participants were nurses, six were senior nurses-in-charge, and two were associate chief nurses. Nine participants were members of the hospital intravenous therapy group (Table 1).

**Table 1:** Demographic characteristics of participants.

ParticipantID	Gender	Age, years	Pediatric nursing experience, years	Educational background	Professional title	Member of the hospital intravenous therapy team	Clinical department	Prior training in vein visualization technology	Prior/current use of vein visualization technology
N1	Female	27	6	Bachelor's degree	Nurse	No	Neurosurgery	No	No
N2	Female	29	7	Bachelor's degree	Nurse	No	Thoracic and oncology surgery	No	No
N3	Female	38	16	Bachelor's degree	Nurse-in-charge	No	General surgery	No	No
N4	Female	42	12	Bachelor's degree	Nurse-in-charge	Yes	General surgery	Yes	Yes
N5	Female	40	11	Postgraduate degree	Associate chief nurse	No	Neurosurgery	Yes	Yes
N6	Female	39	18	Bachelor's degree	Nurse-in-charge	Yes	Cardiovascular medicine	No	Yes
N7	Female	34	12	Bachelor's degree	Nurse-in-charge	Yes	Intensive care medicine	Yes	Yes
N8	Female	29	8	Bachelor's degree	Nurse	No	Neonatal surgery	No	No
N9	Female	43	21	Bachelor's degree	Nurse-in-charge	Yes	Intensive care medicine	Yes	Yes
N10	Female	28	7	Bachelor's degree	Nurse	No	Neonatal medicine	No	No
N11	Female	39	17	Bachelor's degree	Nurse-in-charge	Yes	Hematology	Yes	Yes
N12	Female	30	8	Bachelor's degree	Nurse	Yes	Emergency department	Yes	Yes
N13	Female	39	18	Bachelor's degree	Associate chief nurse	Yes	Neurology	Yes	Yes
N14	Female	32	10	Bachelor's degree	Nurse	Yes	Emergency department	No	No
N15	Female	34	12	Bachelor's degree	Nurse	Yes	Emergency department	Yes	Yes

### 3.2 Overview of identified barriers and facilitators

Using the Theoretical Domains Framework as the analytic framework, barriers and facilitators influencing the implementation of infrared vein visualization technology for pediatric peripheral intravenous catheterization were identified. The findings were mapped to seven TDF domains: knowledge; skills; social/professional role and identity; goals; beliefs about consequences; environmental context and resources; and social influences.

Overall, nine barriers and five facilitators were identified. The barriers included insufficient vascular imaging knowledge, inadequate technical competence, lack of standardized operating procedures, uncertainty regarding professional role boundaries, dependence on conventional cannulation practices, perceived procedural complexity, distrust of device accuracy, lack of pediatric-specific devices, and interference from children's crying and restlessness. The facilitators included enhancement of nurses' professional competence, improved success rate in difficult intravenous access, enhanced puncture efficiency in emergency settings, improved healthcare experience and reduced resource consumption, and positive peer demonstration effect. A summary of the TDF domains, barriers, and facilitators is presented in Table 2.

**Table 2:** TDF-mapped barriers and facilitators to implementing infrared vein visualization technology in pediatric PIVC.

TDF domain	Barriers	Facilitators
Knowledge	1.Insufficient vascular imaging knowledge	—
Skills	2.Inadequate technical competence; 3.Lack of standardized operating procedures	1.Enhancement of nurses’ professional competence
Social/professional role and identity	4.Uncertainty regarding professional role boundaries	—
Goals	5.Dependence on conventional cannulation practices; 6.Perceived procedural complexity;	2.Improved success rate in difficult intravenous access; 3.Enhanced puncture efficiency in emergency settings
Beliefs about consequences	7.Distrust of device accuracy	4.Improved healthcare experience and reduced resource consumption
Environmental context and resources	8.Lack of pediatric-specific devices; 9.Interference from children's crying and restlessness	—
Social influences	—	5.Positive peer demonstration effect

### 3.3 Barriers to the implementation of infrared vein visualization technology

#### 3.3.1 Insufficient vascular imaging knowledge (knowledge)

Nurses widely reported that their knowledge of pediatric vascular imaging was insufficient to support the effective use of vein visualization technology. Pediatric peripheral veins differ from those of adults in that they are smaller in diameter and present unique anatomical challenges, requiring operators to possess microvascular imaging interpretation capabilities. Participants noted that their current understanding of the working principles of imaging devices and the skills needed to interpret vascular images were inadequate.

“Most of our punctures are still performed blindly. We lack adequate understanding of the principles of vascular imaging devices and the techniques for interpreting imaging results.” (N3)

“The anatomical characteristics of children's blood vessels—such as small vessel diameter and thin subcutaneous fat—require operators to have stronger image interpretation abilities and hand–eye coordination. Targeted training has not yet been offered, which increases the difficulty of application.” (N7)

#### 3.3.2 Inadequate technical competence (skills)

Nurses generally reported lacking practical operational skills in using such devices. The primary reason identified was the absence of a structured education and training system for infrared vein visualization technology, leading to fragmented knowledge transfer and reduced technology adoption.

“The current training system has not established admission or credentialing standards for visualization technology. Key dimensions such as device selection criteria, timing of application, and operational protocols all lack systematic guidance.” (N5)

#### 3.3.3 Lack of standardized operating procedures (skills)

Participants highlighted that the standardized operating procedures for intravenous infusion in their hospital did not incorporate a visualization step. As a result, the acquisition of vein visualization skills relied entirely on informal mentoring, with highly variable outcomes. The absence of a standardized protocol contributed to inconsistency in practice and undermined nurses' confidence in applying the technology.

“At present, most of us still perform blind punctures. The standardized operating procedure for intravenous infusion does not include a visualization step. The skills for using the vascular imaging device in our ward can only be acquired through informal peer mentoring, and the results are very inconsistent.” (N9)

#### 3.3.4 Uncertainty regarding professional role boundaries (social/professional role and identity)

Nurses commonly expressed confusion about whether the use of vein visualization technologies fell within the scope of nursing practice. In their accounts, infrared vein visualization and ultrasound-guided vascular access were discussed in close relation: both were perceived as imaging-based techniques that traditionally belong to the domain of radiology and carry significant professional boundaries. The perceived overlap with diagnostic imaging—particularly ultrasound—created role ambiguity and concern about potential scope-of-practice violations among nursing staff.

“Vascular visualization inherently belongs to the field of imaging diagnostics. For example, ultrasound technology has its own professional specificity—would nurses be overstepping their professional boundaries by using it?” (N2)

“Do we need special certification to use ultrasound? I want to use visualization technology to improve puncture success rates, but I’m also worried about the professional liability risks of operating beyond our scope.” (N11)

#### 3.3.5 Dependence on conventional cannulation practices (goals)

A notable barrier identified was nurses’ reliance on established conventional techniques. Senior nurses, in particular, tended to maintain their existing work patterns, expressing the view that blind puncture based on palpation was sufficiently effective in routine cases and that introducing visualization equipment would unnecessarily prolong the procedure without guaranteed improvement in outcomes.

“Blind puncture using palpation to locate blood vessels already has a high success rate in routine cases. Using visualization devices would increase time consumption, and there's still no guarantee of first-attempt success.” (N4)

#### 3.3.6 Perceived procedural complexity (goals)

Many participants perceived the use of infrared vein visualization technology as cumbersome and overly complex, particularly in the pediatric context where the operator must simultaneously manage the device and restrain a moving child. Nurses reported that the additional steps involved in device setup, image focusing, and limb stabilization added to their workload and disrupted the familiar workflow.

“I’m already accustomed to the traditional peripheral venous puncture technique. Now you're asking me to adjust the machine, focus the image on the vessel, and at the same time stabilize the child's puncture site—it just feels very troublesome.” (N8)

#### 3.3.7 Distrust of device accuracy (beliefs about consequences)

Several nurses reported a significant decline in their confidence in infrared vein visualization technology after experiencing situations in which the device's displayed vessel location did not correspond to actual puncture outcomes. Such experiences of perceived inaccuracy eroded trust and led to reluctance to use the equipment in subsequent clinical practice.

“I once really trusted the location it showed, but I failed twice in a row. The child was crying heart-wrenchingly, and the family's expression was awful too. After that, I didn't really want to use it anymore.” (N4)

#### 3.3.8 Lack of pediatric-specific devices (environmental context and resources)

This study revealed that the clinical application of vein visualization technology in pediatric peripheral intravenous catheterization was constrained by the absence of devices specifically designed for children. Children, as a special patient population, exhibit significant age-related variations in venous caliber, depth, and elasticity. These individual differences pose substantial challenges in developing visualization tools that are both precise and practical across different pediatric age groups. Participants reported that currently available devices, though diverse, yielded suboptimal imaging results for children's peripheral veins.

“Although there are many types of visualization tools on the market, their imaging performance for children's peripheral veins is poor. The depth of the vessels is difficult to determine accurately, and the practical value for clinical procedures is limited.” (N10)

#### 3.3.9 Interference from children's crying and restlessness (environmental context and resources)

Beyond device availability, the clinical environment itself posed significant challenges to the effective use of visualization technology. During actual puncture procedures, children frequently cried and moved restlessly, which interfered with the operation of imaging devices and reduced their effectiveness. The complexity of the pediatric clinical environment thus further hindered technology adoption.

“The imaging performance of visualization devices is easily affected by the environment. When children cry and struggle, it makes it difficult to use the tools effectively and also prolongs the puncture time.” (N13)

“The child exhibits crying, excessive limb movements, and inability to cooperate.” (N5).

### 3.4 Facilitators to the implementation of infrared vein visualization technology

#### 3.4.1 Enhancement of nurses' core professional competence (skills)

Nurses widely believed that the high difficulty of pediatric peripheral venous puncture represents a core challenge in pediatric nursing, and that the application of vein visualization technology could directly address this long-standing clinical challenge. This aligned with the broader trajectory of pediatric specialty nursing toward precision and professionalization, allowing nurses to more fully demonstrate the professional value of their role. Participants viewed mastery of visualization technology as an important means of enhancing their professional competence.

“Visualization technology can solve the major problem of difficult venous puncture—it truly embodies the value of specialty nursing.” (N1)

“When nurses master visualization puncture techniques and elevate their intravenous puncture capabilities, it demonstrates core competitiveness. It also provides more opportunities for professional contribution in multidisciplinary collaboration.” (N14)

#### 3.4.2 Improved success rate in difficult intravenous access (goals)

Nurses universally perceived the clinical value of visualization technology, and the anticipated improvement in puncture efficacy and reduction in nursing burden served as a core internal motivator for its use. This was primarily reflected in two aspects: first, the perceived potential to improve the success rate of difficult venous punctures in children, thereby reducing physical and psychological distress; and second, the perceived potential to enhance puncture efficiency in emergency situations, enabling rapid vessel localization during the care of critically ill children.

“This technology can reduce the need for repeated punctures and genuinely alleviate children's suffering.” (N3)

#### 3.4.3 Enhanced puncture efficiency in emergency settings (goals)

In emergency and critical care contexts, nurses perceived that visualization technology could offer distinct advantages by enabling rapid vessel localization, which they considered critical during the resuscitation of critically ill children.

“In emergency situations, pediatric puncture is even more challenging. Visualization technology can help us quickly locate blood vessels and support the resuscitation of critically ill children.” (N12)

#### 3.4.4 Improved healthcare experience and reduced resource consumption (beliefs about consequences)

In the domain of beliefs about consequences, nurses perceived that visualization technology could improve the success rate of difficult venous punctures, thereby reducing nurse–patient conflicts arising from repeated puncture attempts and enhancing the overall healthcare experience for children and their families. Additionally, reducing puncture failure rates could minimize the waste of consumables such as catheters and dressings, lower the costs associated with managing complications, and achieve more efficient use of medical resources.

“A successful puncture can improve understanding from the family and strengthen the foundation of trust between nurses and patients.” (N7)

“Visualization technology can effectively save consumables and medical costs, enabling rational allocation of resources.” (N15)

#### 3.4.5 Positive peer demonstration effect (social influences)

Peer demonstration emerged as an important external motivator for the clinical promotion of visualization technology, reinforcing nurses' willingness and confidence to adopt it. Two key mechanisms were identified: first, the professional demonstration provided by intravenous therapy specialist group members within the hospital, which offered ordinary nurses learning exemplars and fostered a supportive atmosphere for technology adoption within the ward; and second, the sharing of successful cases and practical experiences by peers, which vividly demonstrated the technology's effectiveness, alleviated concerns about its use, and strengthened nurses' confidence in learning and applying it.

“The demonstration role of intravenous therapy experts can help other nurses become proficient in the operation.” (N5)

“Sharing successful cases and technology application experiences can help nurses better appreciate the value of the technology and feel more confident in using it.” (N9)

### 3.5 Summary of overarching themes

The three overarching themes were developed at a higher level of abstraction than the individual codes. Following the mapping of codes to TDF domains, the research team grouped the nine barriers and five facilitators according to the level at which implementation change is required—system, practice, and outcome—through iterative discussion. The resulting themes are described below.

1. System-level implementation conditions require optimization. This theme encompasses organizational-level factors including the need for structured training programs, standardized operating procedures, and adequate provision of pediatric-specific visualization devices.
2. Practice-level willingness among nurses requires enhancement. This theme addresses individual-level factors such as dependence on conventional practices, perceived procedural complexity, professional role uncertainty, and distrust of device accuracy—all of which undermine nurses’ motivation to adopt the technology.
3. Outcome-level sustainability needs strengthening. This theme reflects the need to sustain the use of vein visualization technology over time. It was derived primarily from the facilitators indicating that positive outcome experiences (e.g., perceived improvements in difficult venous access and healthcare experience) and continued peer demonstration could reinforce nurses’ confidence and motivation, thereby supporting the maintenance of the new practice.

## 4 Discussion

This study applied the Theoretical Domains Framework to identify barriers and facilitators to the implementation of infrared vein visualization technology for peripheral intravenous catheterization among pediatric nurses. Across seven TDF domains, nine barriers and five facilitators were identified and synthesized into three overarching themes reflecting system-, practice-, and outcome-level conditions. The findings reveal that the evidence–practice gap in pediatric vein visualization technology implementation is multi-dimensional, involving individual, organizational, and environmental factors.

### 4.1 Knowledge and skills deficits as primary barriers

The present study revealed that insufficient vascular imaging knowledge and inadequate technical competence constituted fundamental barriers to technology adoption. Although the majority of participants were aware that the Infusion Nurses Society (INS) standards recommend the use of visualization technology for children with difficult venous access (1), most nurses reported a lack of practical skills in operating infrared vein visualization devices. This finding is consistent with prior implementation research showing that inadequate training and the absence of structured protocols constitute common barriers to new technology adoption across diverse clinical contexts (15–18).

### 4.2 Professional role boundaries and identity concerns

Uncertainty regarding professional role boundaries emerged as a significant barrier within the social/professional role and identity domain. Nurses expressed concern that the use of vein visualization technologies might exceed the scope of nursing practice. Participants discussed both infrared vein visualization and ultrasound-guided vascular access and perceived both as imaging-based techniques that traditionally belong to the domain of radiology. Because ultrasound guidance is the more established imaging-based modality in vascular access, the perceived overlap with diagnostic imaging—and the associated scope-of-practice concerns—were particularly salient for ultrasound, and this concern extended to infrared vein visualization as well. This finding suggests that clarifying the nature and scope of the specific technology during training and implementation may be as important as the device itself. This finding suggests that clarifying the nature and scope of the technology during training and implementation may be as important as the device itself. This finding parallels those of Debono et al. (17), who identified professional role conflict as a major barrier to nurses' use of electronic medication management systems, and Kirk et al. (18), who reported that professional boundaries were perceived as barriers to implementing screening tools in emergency departments. In both studies, as in the present study, practitioners set up boundaries to protect their established work practices when confronted with new technologies that appeared to challenge their professional identity.

These findings suggest that in the Chinese healthcare context, where hierarchical clinical-nursing dynamics remain pronounced (19), the introduction of imaging-based technologies into nursing practice requires explicit clarification of scope-of-practice boundaries. Without such clarification, nurses may experience role ambiguity that inhibits technology adoption, even when the technology is evidence-based and clinically beneficial.

### 4.3 Goals and behavioral inertia

Three barriers were identified within the Goals domain: dependence on conventional cannulation practices, perceived procedural complexity, and distrust of device accuracy. The persistence of traditional blind-puncture techniques among experienced nurses aligns with well-established findings in implementation science that habitual behavior and behavioral inertia constitute significant barriers to practice change (11). Senior nurses, in particular, expressed the view that their existing techniques were sufficiently effective in routine cases, rendering the adoption of new technology unnecessary.

Of particular interest is the emergent barrier of distrust of device accuracy, which has rarely been reported in prior literature on vein visualization technology. Several nurses in the present study described negative experiences in which the device's displayed vessel location did not correspond to actual puncture outcomes, leading to repeated failed attempts and emotional distress. These negative outcome expectations shaped a lasting belief that the device was unreliable, resulting in technology avoidance. This finding is noteworthy in light of the study by Szmuk et al. (20), a randomized controlled trial involving 600 pediatric patients, which found that the VeinViewer vascular imaging system actually worsened the first-attempt cannulation rate for experienced nurses (47% vs. 62% in the standard group). While Szmuk et al. attributed this paradox to nurses’ over-reliance on the visual image at the expense of other cues, the present qualitative findings suggest an additional mechanism: the erosion of trust following negative experiences. Together, these findings highlight that device accuracy is not solely a technical issue but also a psychological one, with implications for how technology is introduced, monitored, and supported in clinical settings.

### 4.4 Environmental and resource constraints

The lack of pediatric-specific visualization devices and interference from children's crying and restlessness were identified as key barriers within the environmental context and resources domain. These findings are consistent with the mixed evidence in the literature regarding the effectiveness of near-infrared devices in pediatric populations. A systematic review and meta-analysis by Kuo et al. (21) of 12 studies concluded that near-infrared devices did not significantly improve first-attempt success rates, number of attempts, or procedural time in the general pediatric population. Similarly, Conversano et al. (22) found no benefit of routine VeinViewer use in children across three age subgroups. These findings suggest that current devices may not be adequately optimized for the pediatric population, corroborating the present study's finding that nurses perceived existing tools as having suboptimal imaging performance for children's peripheral veins.

### 4.5 Facilitators: positive beliefs, professional development, and peer influence

Nurses recognized that vein visualization technology could enhance their professional competence by addressing a long-standing clinical challenge in pediatric vascular access. This finding aligns with the growing emphasis on specialty nursing development and the pursuit of precision care in pediatric settings, where vein visualization technology has been recommended by multiple professional organizations including the Emergency Nurses Association, the Association for Vascular Access, and the Infusion Nurses Society (23).

Within the beliefs about consequences domain, nurses expressed positive expectations that the technology could improve the healthcare experience for children and families, reduce nurse–patient conflicts, and conserve medical resources by minimizing consumable waste. These positive outcome expectations stand in contrast to the negative beliefs arising from device inaccuracy experiences described above, suggesting that beliefs about consequences function as a double-edged sword: positive experiences reinforce adoption, whereas negative experiences erode confidence. This duality highlights the critical importance of ensuring device reliability and providing adequate initial training to prevent negative first experiences that may have lasting deterrent effects.

Within the goals domain, the perceived ability of visualization technology to improve difficult venous puncture success rates and enhance emergency puncture efficiency served as strong intrinsic motivators. These findings are supported by clinical evidence: studies by Çağlar et al. (24) and Demir and Inal (25) demonstrated that infrared vein visualization devices reduced the number of puncture attempts and procedural time while increasing first-attempt success rates in pediatric populations. Together, the available clinical evidence and nurses' positive perceptions suggest that targeted rather than routine use of the technology may be beneficial.

Finally, the positive peer demonstration effect within the social influences domain emerged as an important external facilitator. Intravenous therapy specialist group members served as clinical champions whose professional demonstrations motivated other nurses to adopt the technology. The role of clinical champions in facilitating evidence implementation is well documented (11, 26), and in this study peer-led demonstrations and success-sharing appeared particularly effective in overcoming initial skepticism and building collective confidence. To support long-term sustainability, nursing administrators may also consider establishing ongoing quality monitoring mechanisms (e.g., periodic review of device use and outcomes, and structured feedback to nursing staff). This recommendation is grounded in the present findings but was not directly explored in the data and requires feasibility testing and evaluation in future research.

## 5 Limitation

Several limitations should be acknowledged. First, the study was conducted at a single tertiary children's hospital in Beijing, and the 15 participants were relatively homogeneous in terms of education level (all holding bachelor's degrees) and clinical setting. The findings may not be transferable to primary or community healthcare settings, or to hospitals in other regions with different resource configurations. Second, the sample consisted exclusively of nurses with at least five years of pediatric experience; the perspectives of novice nurses or nursing students, who may have different training needs and technology adoption patterns, were not captured. Third, as a qualitative study, the findings represent nurses' perceived barriers and facilitators—including perceived clinical benefits of the technology—rather than objectively measured outcomes; outcomes such as improved first-attempt success or reduced resource consumption were not quantitatively evaluated. Fourth, the study did not examine patients' or parents' perspectives, which may provide complementary insights into the acceptability and perceived value of vein visualization technology.

## 6 Conclusion

The clinical application of infrared vein visualization technology for peripheral intravenous catheterization in pediatric patients is influenced by multiple interrelated factors spanning seven TDF domains. While the technology's clinical value is broadly recognized by nurses, its translation into routine practice is hindered by knowledge and skills deficits, professional role uncertainty, behavioral inertia, distrust of device accuracy, lack of pediatric-specific equipment, and contextual challenges unique to pediatric care. Conversely, enhancement of professional competence, perceived clinical benefits, positive outcome expectations, and peer demonstration effects serve as important facilitators. These findings may inform targeted, theory-informed implementation strategies that address the identified barriers while leveraging the facilitators, with the aim of bridging the evidence-practice gap.

## Data Availability

The original contributions presented in the study are included in the article/Supplementary Material, further inquiries can be directed to the corresponding author/s.
