# Supplementary material for: What barriers and facilitators influence the implementation of infrared vein visualization technology for pediatric peripheral intravenous catheterization? A TDF-informed qualitative study
Source: Front Pediatr. 2026 Oct 29;14:1933634. doi: 10.3389/fped.2026.1933634 (PMC13755256; doi:10.3389/fped.2026.1933634)
Supplement: Supplementary file 1 [file Table1.docx]

Supplementary Table S1. Semi-structured interview guide

Module 1.

*Corresponding TDF domains: Knowledge; Skills; Memory, attention and decision processes.*

1.What knowledge and skills do you think are required to use an infrared vein visualization device proficiently?

In actual clinical practice, what do you consider to be the greatest technical difficulty?

2.Do you think there is any conflict between identifying veins on the screen and palpating veins by hand?

How do you allocate your attention during the procedure?

Do you ever feel that focusing on one aspect may cause you to neglect another?

3.When caring for a child with extremely poor vascular conditions, how do you decide when to use the device and when to abandon the device and proceed with conventional or blind cannulation?

Is this decision mainly based on clinical intuition, or are there clear criteria guiding your decision?

Module 2.

*Corresponding TDF domains: Emotion; Optimism; Beliefs about consequences; Social/professional role and identity.*

1.What are your psychological or emotional experiences when using an infrared vein visualization device for pediatric intravenous catheterization?

2.To what extent do you think this technology can improve the current situation of pediatric intravenous catheterization?

Are you optimistic about its future application in pediatric clinical practice?

3.Do you think the use of this device has any influence on your professional role identity as a pediatric nurse?

If yes, in what way?

Module 3.

*Corresponding TDF domains: Environmental context and resources.*

1.Has your department organized any specific training on the use of infrared vein visualization technology?

How effective was the training?

Are there any assessment, supervision, or quality-control standards related to its use?

2.In your opinion, does using an infrared vein visualization device increase your workload, or does it simplify the workflow? Could you give an example?

3.In your view, should this technology be used routinely, or should it be used only as a rescue measure after failed cannulation attempts? Why?

Module 4.

*Corresponding TDF domains: Social influences.*

1.What are the attitudes of your leaders, colleagues, and physicians toward the use of infrared vein visualization technology?

2.Do their attitudes influence your willingness to use the device?

Do nurses in your department share experiences or practical tips regarding its use?

3.What are the opinions or reactions of children’s family members when this device is used?

4.How do family members’ attitudes influence your psychological state, procedural confidence, or clinical decision-making?

Module 5.

*Corresponding TDF domains: Goals; Intentions; Reinforcement; Behavioral regulation.*

1.What is your main purpose when using an infrared vein visualization device?

2.What factors would encourage you to continue using it actively and appropriately in the future?

3.To use the device more effectively, or when encountering difficulties during use, have you taken any measures yourself?

For example, have you sought training, consulted colleagues, summarized experience, or adjusted your technique?

Module 6. Summary and additional comments

*Corresponding TDF domains: Open-ended; not tied to a specific TDF domain.*

1.Overall, what do you think is the most important facilitator that currently encourages you to continue using infrared vein visualization technology in pediatric intravenous catheterization?

2.What is the most critical barrier that makes you unwilling or unable to use it?

3.If you could make one recommendation to the hospital or department to help this technology better serve pediatric patients, what would you suggest?

4.Is there anything important that we have not discussed but that you would like to add?
